# The Bidirectional Role of Cellular Senescence in the Treatment of Ovarian Cancer

**DOI:** 10.3390/biomedicines14081797

**Published:** 2026-08-10

**Authors:** Didi Yuan, Mengying Chen, Jinmei Wei, Yang Zhang, Yanling Li, Ning Tang, Chen Han, Yanhong Zhou

**Affiliations:** 1 Department of Radiation Oncology, Hunan Cancer Hospital, Central South University, Changsha 410013, China; 2 Cancer Research Institute, Basic School of Medicine, Central South University, Changsha 410011, China; 3 Medical Record Management and Information Statistics Center, Xiangya Hospital, Central South University, Changsha 410011, China; 4 Department of Nuclear Medicine, Hunan Cancer Hospital, Central South University, Changsha 410013, China; 5 Office of Scientific Research Administration, Hunan Cancer Hospital, Central South University, Changsha 410013, China

**Keywords:** ovarian cancer, cellular senescence, treatment, bidirectional effect, treatment target

## Abstract

Cellular senescence plays a complex and crucial dual role in ovarian cancer treatment: it serves as both an important tumor suppression mechanism and a potential driver of drug resistance and recurrence. This review systematically examines the intricate regulatory network of cellular senescence, encompassing multiple pathways including telomere dysfunction, DNA damage response, epigenetic remodeling, hormone signaling, and metabolic reprogramming. Studies have demonstrated that core therapeutic modalities, such as platinum-based chemotherapy, PARP inhibitors, and CDK4/6 inhibitors, can exert anti-tumor effects by inducing cellular senescence; however, their efficacy is significantly influenced by the senescence-associated secretory phenotype (SASP). Specific SASP components can activate immune surveillance, whereas others promote tumor progression, acquisition of stem cell-like characteristics, and therapeutic resistance. It is this “anti-tumor versus pro-tumor” paradox that renders senescent cells a critical variable determining treatment outcomes. In light of this, emerging strategies aim to precisely modulate the senescence process, including the use of epigenetic drugs to induce senescence, targeting metabolic vulnerabilities to eliminate senescent cells, and intervening in deleterious SASP to improve the tumor microenvironment. Integrating such strategies with conventional therapies holds promise for overcoming the resistance bottleneck in ovarian cancer and opening new avenues for improving patient prognosis.

## 1. Introduction

Cellular senescence is traditionally defined as a stable state of proliferative arrest accompanied by characteristic alterations in cellular morphology, metabolism, chromatin organization, and the secretion of senescence-associated secretory phenotype (SASP) factors [1]. The concept of irreversible cell-cycle withdrawal represents a fundamental feature distinguishing senescence from transient growth inhibition. However, accumulating evidence indicates that senescence, particularly therapy-induced senescence (TIS) in cancer, should not be considered a uniform and permanently fixed endpoint. Unlike physiological senescence, TIS is induced by diverse therapeutic stresses, including chemotherapy, targeted therapy, and radiotherapy, and its biological stability varies depending on treatment intensity, tumor genetic background, and microenvironmental context. Some therapy-induced senescent cancer cells undergo durable proliferative arrest and can be eliminated by immune surveillance, thereby contributing to tumor control [2,3]. Conversely, a subset of senescent cells may retain phenotypic plasticity and acquire the ability to escape senescence through cell-cycle re-entry, stemness acquisition, or alternative proliferation mechanisms, such as polyploid giant cancer cell-mediated reproduction [4]. Therefore, cellular senescence should be distinguished from other states of reduced proliferation, including quiescence, which represents a reversible resting state; dormancy, characterized by long-term tumor cell persistence with limited proliferative activity; transient treatment-induced cell-cycle arrest; and drug-tolerant persister states associated with reversible therapeutic adaptation.

The core mechanisms of ovarian cancer cell senescence primarily involve two major pathways: telomere-dependent pathways (including telomere shortening and alterations in telomerase activity) and non-telomere-dependent DNA damage response pathways [5,6]. Epigenetic regulation—including DNA methylation, histone modifications, and the function of non-coding RNAs—plays a profound role in the initiation and maintenance of the cellular senescence program [7,8,9]. Additionally, hormonal levels constitute critical regulatory factors, particularly estrogen and progesterone, which profoundly influence the senescence process of ovarian cancer cells through their complex interactions and dynamic equilibrium with their respective receptors [10,11]. Recent studies have further demonstrated that metabolic stress represents an intrinsic key factor driving ovarian cancer cells into senescence [12,13,14]. In summary, the regulatory network governing ovarian cancer cell senescence is exceedingly complex. Comprehensive analysis of the interaction network woven by these factors is crucial for clarifying the pathological mechanisms of ovarian cancer and developing novel therapeutic strategies, such as inducing senescence or targeting the clearance of senescent cells.

Ovarian cancer cell senescence plays a complex dual role in tumor proliferation, migration, drug resistance, and prognosis. Studies have demonstrated that various chemotherapeutic agents (including carboplatin and paclitaxel) and targeted therapies (including olaparib) can induce G1 or G2/M phase cell cycle arrest, upregulate key proteins such as p21 and p53, thereby triggering cancer cell senescence and inhibiting proliferation [15,16]. However, senescent cells are not completely inert. They secrete senescence-associated secretory phenotype (SASP) factors—comprising various cytokines, chemokines, and growth factors—which stimulate the proliferation, migration, and epithelial mesenchymal transition (EMT) of surrounding non-senescent cancer cells in a paracrine manner, thereby promoting tumor progression and recurrence [15]. With respect to drug resistance, cellular senescence exhibits a “double-edged sword” effect: on the one hand, inducing senescence represents a critical mechanism by which chemotherapeutic agents inhibit tumor growth; on the other hand, senescent cells can acquire resistance to apoptosis by activating specific signaling pathways such as Aurora-A/SOX8/FOXK1 and NAC1/GADD45GIP1, thereby mediating resistance to platinum-based drugs and PARP inhibitors [16,17]. From a prognostic perspective, successful induction of senescence at the early stage of treatment is typically associated with favorable chemotherapy response. However, if senescent cells persist, the SASP produced by these cells will shape an inflammatory and immunosuppressive tumor microenvironment [18], ultimately leading to disease recurrence and poor prognosis.

Currently, research on ovarian cancer cell senescence has shifted toward reversing chemotherapy resistance. In this context, the strategy of combining senolytic agents with chemotherapy or targeted therapy has demonstrated significant synergistic effects in preclinical models [19,20]. The development and validation of this strategy are based on a profound understanding of the dual roles of cellular senescence. This review systematically explores various factors that induce senescence in ovarian cancer cells and focuses on the dual roles of senescence in treatment. Building upon these insights, researchers are actively advancing the translational application of targeted senescence therapy. For example, inhibiting NAMPT or ALDH1A1 eliminates senescence-associated cancer stem cells, thereby sensitizing tumors to platinum-based chemotherapy [21,22]. Additionally, innovative therapeutic approaches are being explored that combine senolytic agents or SASP modulators with immunotherapy or ferroptosis inducers [23,24]. These cutting-edge investigations provide direction for the precise utilization and management of ovarian cancer cell senescence in clinical practice.

## 2. Telomere-Related Ovarian Cancer Cell Senescence

Compared with normal ovarian tissue, ovarian cancer tissue exhibits not only shorter telomere lengths but also significantly elevated telomerase activity [25,26]. Although no significant difference in telomere length exists between sporadic and hereditary ovarian cancer, both demonstrate significantly shorter telomeres than normal tissue [25]. While telomere shortening itself is not affected by homologous recombination deficiency, the coexistence of both may synergistically promote the early onset of ovarian cancer [25]. Telomerase reactivation represents a key feature of ovarian cancer, and its activity is closely associated with poor patient prognosis. Studies have demonstrated that telomerase is expressed in 95% of gynecological malignancies and 88% of epithelial ovarian cancers, whereas its activity is extremely low or undetectable in most normal somatic tissues [27]. In ovarian tumors, telomerase activity is highly consistent with mRNA expression levels of human telomerase reverse transcriptase (hTERT) (approximately 80%) [28]. hTERT mRNA expression exhibits significant heterogeneity among different ovarian cancer subtypes, between benign and malignant tumors, and even among patients with the same histological type [29]. This differential expression likely stems from variations in promoter activity [28,30]. Within the core promoter region 283 bp upstream of the hTERT transcription start site (TSS), two regulatory elements, MT-box and E-box, have been identified, and both jointly drive telomerase activation [28]. RIF1 can directly bind to the hTERT promoter to upregulate its expression [31].

Inhibition of telomerase function can exert anti-ovarian cancer effects through two parallel pathways: endoreduplication-dependent senescence and activation of non-endoreduplication-dependent rapid apoptosis. However, the causal relationship between telomerase activity and telomere length maintenance in ovarian cancer remains controversial. For instance, in telomerase-positive SKOV-3 cells, even complete inhibition of telomerase function does not significantly affect telomere maintenance mechanisms [32]. Similarly, nuclear enzyme-mediated telomerase inhibition can directly induce apoptosis in ovarian cancer cells without causing telomere shortening [33]. In contrast, cell models treated with hTERT antisense oligonucleotides exhibit typical telomerase-dependent telomere shortening and subsequent cellular senescence [34]. Further mechanistic studies have confirmed that hTERT downregulation can promote p53 and p21 expression to induce senescence and directly trigger apoptosis in p53-mutated ovarian cancer cells [35]. These studies collectively demonstrate that telomerase inhibition can induce cellular senescence through telomere-dependent pathways and directly trigger apoptosis through non-telomere-dependent rapid pathways.

## 3. Epigenetic Regulation-Related Ovarian Cancer Cell Senescence

**Figure 1:**
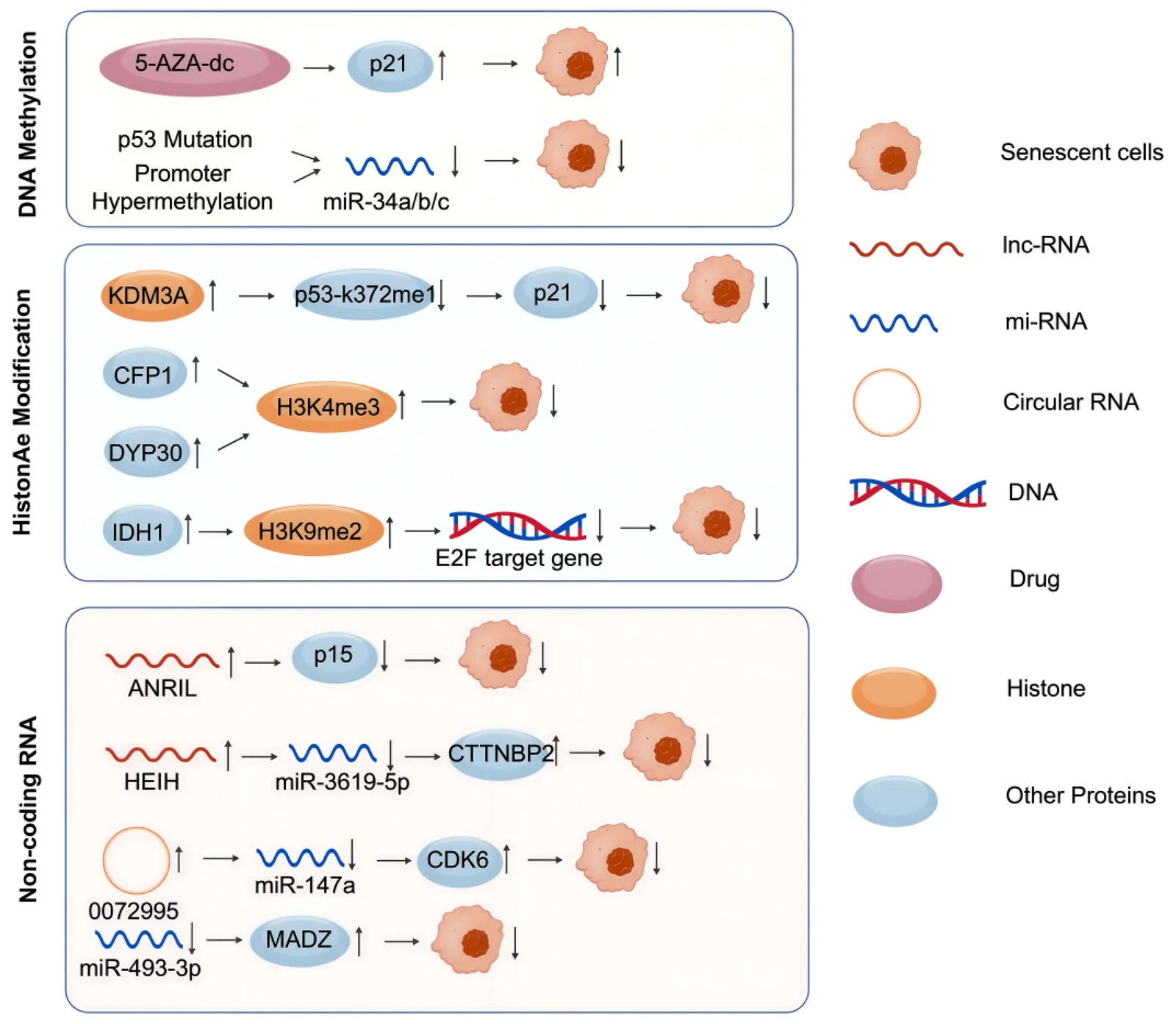
Network illustrating epigenetic modifications in ovarian cancer cell senescence regulation. Schematic summarizing key epigenetic mechanisms—DNA methylation, histone modifications, and non-coding RNAs—that modulate tumor suppressor gene expression and cellular senescence in ovarian cancer. The DNA methyltransferase inhibitor 5-AZA-dC induces p21 expression, whereas promoter hypermethylation of miR-34a/b/c correlates with p53 mutation status. Histone modifiers, including KDM3A, DPY30, and IDH1, regulate p53-K372me1, H3K4me3, and H3K9me2 marks to influence E2F target genes and senescence. Non-coding RNAs, such as ANRIL, miR-3619-5p, and circ_0072995, form competing endogenous RNA networks that control p15, CTTNBP2, and CDK6 expression. Molecular types are color-coded as indicated in the legend. This diagram illustrates the multi-layered epigenetic crosstalk underlying therapy resistance and senescence evasion in ovarian cancer.

DNA methylation represents a common epigenetic inactivation mechanism in ovarian cancer, directly leading to silencing of tumor suppressor genes and resistance of ovarian cancer cells to senescence. Studies have confirmed that miR-34a and miR-34b/c family members, as important tumor suppressor miRNAs, are downregulated in ovarian cancer and other malignancies due to hypermethylation of promoter CpG islands [7] (Figure 1). As a downstream target of p53, low miR-34a expression is associated with p53 mutation status [36]. However, whether DNA methylation and p53 interact in regulating miR-34a expression and senescence resistance requires further investigation. Additionally, the DNA methyltransferase inhibitor 5-AZA-dC has been found to induce expression of the cellular senescence marker p21 while concurrently altering glycosylation patterns in chemotherapy-sensitive cell lines and enhancing EMT and migratory capacity, revealing the complexity of epigenetic therapeutic effects [37].

Histone modifications profoundly influence gene expression and fate determination in ovarian cancer cells by altering chromatin states. EZH2 (which catalyzes H3K27me3) is highly expressed in ovarian cancer, and its expression level is positively correlated with FIGO stage and grade but negatively correlated with tumor suppressor gene p53 expression. This suggests that EZH2 may promote senescence resistance and tumor progression by inhibiting the p53 pathway [8]. KDM3A (a histone demethylase) can downregulate p21 and Bcl-2 expression through demethylation of p53-K372me1, thereby inhibiting cellular senescence and apoptosis [38]. Another classic histone demethylase, JMJD3, has also been reported to participate in the generation of ovarian cancer stem cells and senescence resistance [39]. Additionally, DPY30 and CFP1, as key components of the histone H3K4 trimethylation (H3K4me3) complex, promote ovarian cancer cell proliferation and invasion while inhibiting senescence when highly expressed [40]. Conversely, the histone deacetylase inhibitor trichostatin A can induce ovarian cancer cell cycle arrest and senescence-like differentiation by upregulating p21 and reducing Rb protein phosphorylation [41]. Recent studies have demonstrated that IDH1 affects energy metabolism in high-grade serous carcinoma (HGSC) and increases repressive histone H3K9me2 marks at multiple E2F target gene loci, revealing the mechanism by which metabolic adaptation induces senescence resistance through epigenetic modulation [42].

Various non-coding RNAs play crucial roles in regulating ovarian cancer cell senescence. High expression of the long non-coding RNA ANRIL inhibits senescence and apoptosis by downregulating p15INK4B and upregulating Bcl-2 [43]. HEIH functions as a sponge for miR-3619-5p, accelerating tumor progression and promoting senescence resistance by relieving its inhibition of CTTNBP2 [44]. In contrast, LINC-PINT and sin-lncRNA have been demonstrated to possess tumor-suppressive functions that induce or maintain senescence [45,46]. Among microRNAs, miR-493-3p disrupts the mitotic checkpoint by targeting MAD2, leading to genomic instability and inducing senescence while also conferring resistance to paclitaxel [47]. miR-147a is sequestered by circ_0072995, thereby relieving its inhibition of CDK6 and promoting tumor development [48]. miR-34a/b/c represent key senescence-inducing factors regulated by p53, and their inactivation is common in ovarian cancer [7]. Additionally, the TP63/miRNA network is recognized as crucial in regulating cell cycle, apoptosis, and senescence [49].

As a post-transcriptional modification, N6-methyladenosine (m^6^A) RNA methylation plays a significant role in senescence-related signaling pathways in ovarian cancer cells. Yin et al. constructed an independent prognostic prediction model for ovarian cancer using three m^6^A regulatory factors (IGF2BP1, VIRMA, and ZC3H13). Gene set enrichment analysis indicated that these factors were significantly associated with tumor-related pathways and the Wnt/β-catenin signaling pathway, suggesting that m^6^A modification influences ovarian cancer cell senescence by regulating key signaling pathways [50].

## 4. Hormone-Related Ovarian Cancer Cell Senescence

**Figure 2:**
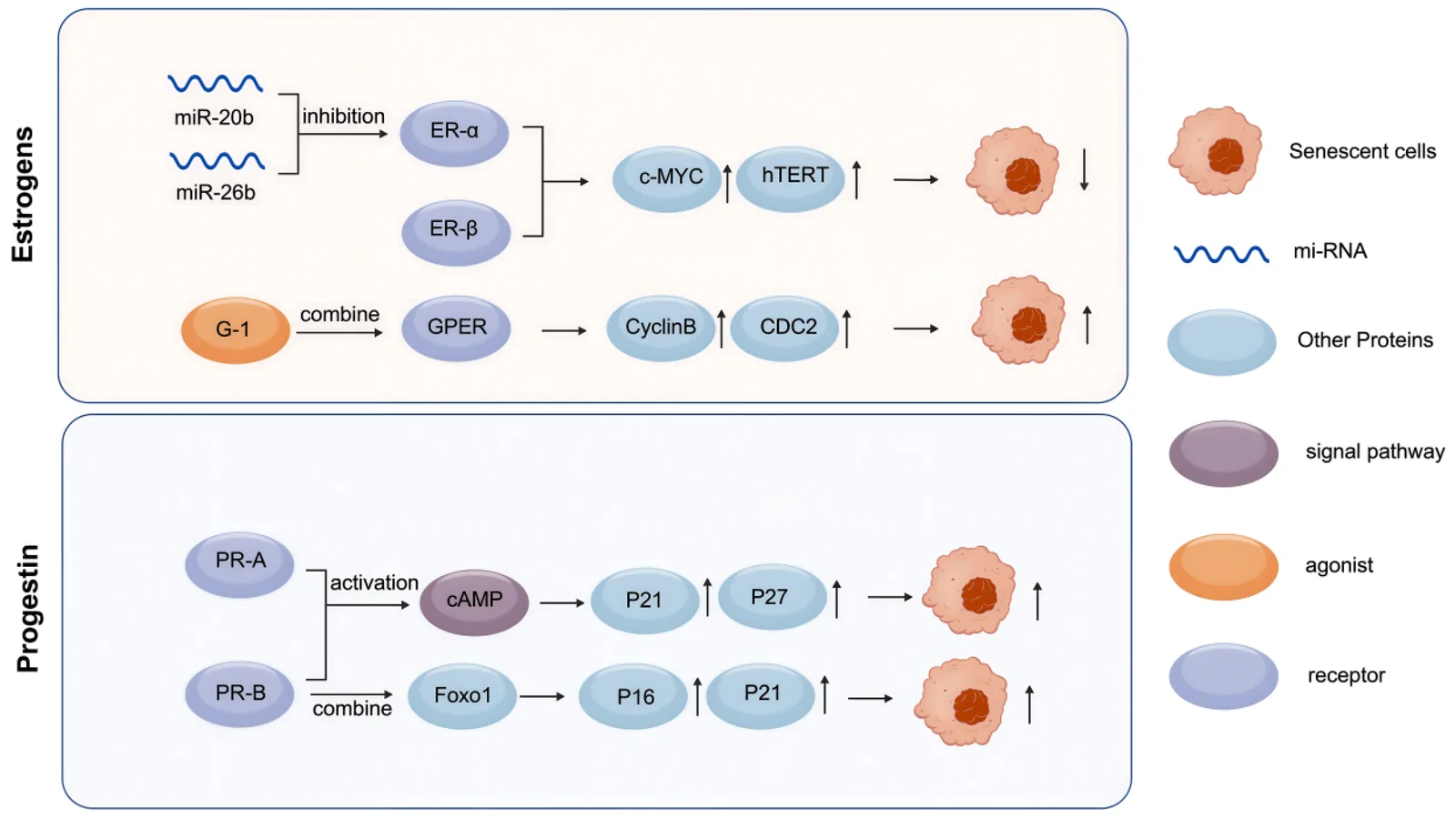
Schematic diagram of hormone regulation of ovarian cancer cell senescence. Estrogens modulate senescence via the ERα/ERβ–c-Myc–hTERT axis, with miR-206 and miR-26b involved in ER crosstalk. The GPER agonist G-1 induces Cyclin B/CDC2 expression. Progestins act through PR-A (↑cAMP→p21/p27) and PR-B (↑FOXO1→p15/p16/p21/p27). This network highlights steroid hormone–receptor–senescence signaling axes.

Estrogen exhibits complex bidirectional regulation during the senescence process of normal and tumor tissues. Estrogen deficiency in young, healthy mice induces telomere shortening in granulosa cells and ovarian senescence. Studies have demonstrated that this telomere-dependent senescence is closely associated with downregulation of the proto-oncogene c-Myc and human telomerase reverse transcriptase (hTERT), and that estrogen replacement therapy effectively reverses the ovarian senescence phenotype by upregulating c-Myc and hTERT expression [51]. Given the crucial role of estrogen in maintaining telomere stability, its excessive activity may continuously activate c-Myc and telomerase, thereby conferring cellular senescence resistance and driving tumorigenesis. Clinical evidence supports this hypothesis: elevated estrogen levels have been detected in the serum of postmenopausal women with epithelial ovarian cancer [52]. Conversely, experimental evidence from non-ovarian cancer models has suggested that estradiol (E2) can also modulate mitochondrial autophagy-related pathways and senescence-associated markers, leading to the induction of senescence phenotypes in certain cellular contexts [53]. Dehydroepiandrosterone (DHEA) treatment has also been found to significantly enhance SA-β-gal activity in KGN cells and primary rat granulosa cells through activation of the SIRT1/p53/caspase-3 signaling pathway [54] (Figure 2). These studies collectively demonstrate that estrogen regulates cellular senescence in a context-dependent manner.

Estrogen achieves bidirectional regulation of ovarian cancer cell senescence through distinct receptor types. These receptors primarily comprise classical nuclear receptors (ERα and ERβ) and the non-classical membrane receptor GPER1 (G protein-coupled estrogen receptor) [11]. Expression of these receptors is precisely regulated. For instance, miR-206 and miR-26b can downregulate ERα protein levels by targeting ESR1 mRNA [55]; additionally, polymorphisms and linkage disequilibrium of SNP loci within the ESR1 gene (such as rs2234693 and rs9340799) significantly influence its expression [56]. Functionally, ERα and ERβ often exhibit antagonistic effects. For example, ERβ inhibits cyclin D1 expression, whereas ERα promotes it [57]. This functional divergence is also reflected in clinical prognosis. Both high ERα and ERβ expression are associated with prolonged overall survival, with the prognostic significance of ERα being particularly pronounced [58]. The non-classical receptor GPER1 also participates in regulatory processes, and its specific agonist G-1 can upregulate Cyclin B and CDC2 expression, thereby inhibiting cell cycle progression in ovarian cancer cells [59]. These findings collectively demonstrate that the composition and balance of the estrogen receptor network are critical determinants of whether estrogen ultimately outputs pro-survival or pro-senescence signals in ovarian cancer.

Progesterone and its receptor (PR) exert tumor-suppressive functions in ovarian cancer by inducing cellular senescence. Notably, the biological effects of progesterone are tissue-specific: it promotes differentiation and proliferation in breast tissue, whereas it significantly inhibits estrogen-driven growth in reproductive tracts and ovaries. Clinical data demonstrate that PR expression serves as a favorable biomarker for ovarian cancer prognosis, with PR-positive patients exhibiting significantly better progression-free survival than PR-negative patients [60]. Studies have shown that progesterone can promote expression of cell cycle arrest factors p21 and p27 by activating cAMP, or induce ovarian cancer cell senescence through a FOXO1-dependent pathway [60,61]. Mechanistic studies have revealed that only PR-B can form a functional complex with the transcription factor FOXO1 and be specifically recruited to promoter regions of target genes such as p15, p16, p21, and p27, thereby robustly driving the cellular senescence program by reshaping active chromatin structure. Recent research has further expanded the mechanisms of progesterone action: first, it can enhance irinotecan sensitivity by inhibiting topoisomerase I (Topo I) [62]; second, it can enhance niraparib efficacy by inhibiting GPX4 and promoting lipid peroxidation-mediated ferroptosis [63].

## 5. Drug-Related Ovarian Cancer Cell Senescence

In ovarian cancer treatment, both chemotherapeutic agents and targeted therapies do not merely induce cell death—they also function as potent senescence inducers, capable of forcing cancer cells to exit the cell cycle. A thorough understanding of the core mechanisms underlying drug-induced cellular senescence is essential for transforming challenges into opportunities in this field.

### 5.1. Chemotherapy

Platinum-based chemotherapeutic agents constitute the fundamental treatment regimen for ovarian cancer [64]. These agents trigger severe DNA damage, thereby activating the core p53-p21 signaling pathway and ultimately inducing G1/S or G2/M phase cell cycle arrest in ovarian cancer cells, accompanied by SASP factor release [65,66]. This senescence induction phenomenon has been extensively validated across various ovarian cancer cell models, including SKOV3, OVCAR3, A2780, and their corresponding drug-resistant sublines [67].

Notably, significant differences exist in the specific mechanisms of action among distinct platinum-based agents. GRP78 has been identified as a critical node regulating cisplatin-induced senescence and drug resistance in ovarian cancer cells. Studies have demonstrated that GRP78 staining is relatively weak in chemotherapy-sensitive ovarian cancer tissues. In cell models, cisplatin-sensitive A2780 cells exhibit low GRP78 expression and heightened susceptibility to senescence, whereas drug-resistant C13K cells display elevated GRP78 levels. Functionally, forced overexpression of GRP78 protects A2780 cells from cisplatin-induced senescence by modulating the p53 and CDC2 pathways [6]. The combination of carboplatin and paclitaxel induces senescence in primary high-grade serous ovarian cancer cells by activating STAT3 signaling and enhancing oxidative stress levels [6,15]. Conversely, oxaliplatin promotes senescence phenotype formation by downregulating ZEB1 and interfering with the Wnt/β-catenin signaling pathway [68]. Additionally, evidence suggests that oxaliplatin and cisplatin may exhibit context-dependent differences in inducing stress ligand expression and regulating immunogenicity [69]. Further studies have confirmed that oxaliplatin participates in remodeling the senescence-associated tumor microenvironment by regulating matrix metalloproteinase expression (such as MMP-2 and MMP-9) [66]. This indicates that the senescence microenvironment shaped by distinct platinum-based agents may possess unique characteristics.

Furthermore, chemotherapy-induced senescent tumor cells can express stemness-related markers. Studies have demonstrated that in a p53-deficient ovarian cancer cell model, the senescent cell subpopulation induced by carboplatin exhibits typical stem cell-like characteristics [70]. Moreover, polyploid giant cancer cells have been identified in doxorubicin-induced senescent ovarian cancer cells. These cells display typical senescent characteristics while highly expressing key stem cell markers such as OCT4 and KLF4, presenting a unique hybrid “senescence-stemness” phenotype [71].

### 5.2. PARP Inhibitor

PARP inhibitors can induce DNA damage-dependent senescence in ovarian cancer cells. Studies have demonstrated that continuous low-dose olaparib treatment can trigger senescence through p16- or p53-dependent pathways, or through the p21 pathway in a p53-independent manner [16,19,72]. Moreover, olaparib can exacerbate DNA damage and promote senescence by upregulating miR-125a-3p, thereby further enhancing tumor cell sensitivity to chemotherapy [73]. Notably, the senescence-promoting effect of PARP inhibitors is broad-spectrum and not restricted to tumors with homologous recombination deficiency. These agents can also effectively induce senescent phenotypes and accompanying SASP in homologous recombination-proficient cancer cells [74].

The senescent state induced by PARP inhibitors is reversible. Studies have demonstrated that tumor cells in senescence can re-enter the cell cycle and resume proliferation upon drug withdrawal [19]. This phenomenon provides a potential biological rationale for the clinical strategy of continuous administration. Further mechanistic studies revealed that treatment induces downregulation of the long non-coding RNA PART1, which promotes degradation of the binding protein PHB2, thereby impairing mitochondrial autophagy and driving cells into a senescence state associated with drug resistance, ultimately resulting in cross-resistance to PARP inhibitors and cisplatin [75]. To address this challenge, researchers proposed an innovative therapeutic strategy: first, utilizing PARP inhibitors to induce tumor cell senescence, followed by combination with specific agents for senescent cell clearance. This strategy has demonstrated significant synergistic anti-tumor effects in preclinical models, opening new avenues to overcome senescence-mediated therapeutic resistance [75].

### 5.3. CDK4/6 Inhibitor

Cyclin-dependent kinase 4/6 (CDK4/6) constitutes a key regulatory factor driving the G1-to-S phase transition of the cell cycle. Its inhibitor, palbociclib, primarily induces ovarian cancer cell senescence through two mechanisms: inhibition of retinoblastoma (Rb) protein phosphorylation and promotion of p53 acetylation [76]. Another CDK4-targeted inhibitor, CDDD2-94, can also effectively induce G1/G0 phase cell cycle arrest and senescence. Compared with palbociclib, CDDD2-94 exhibits reduced hematological toxicity [77].

CDK4/6 inhibitors demonstrate synergistic efficacy in combination and maintenance therapeutic settings. In advanced high-grade serous ovarian cancer models, the regimen comprising ribociclib in combination with cisplatin followed by ribociclib maintenance therapy exhibited superior anti-tumor efficacy. Its mechanism of action extends beyond inducing G1 phase cell cycle arrest; it can also enhance the cytotoxicity of DNA-damaging agents (such as cisplatin) by interfering with the ATR-CHK1-mediated G2/M checkpoint [78]. Furthermore, CDDD2-94 generates synergistic anti-proliferative effects with various classes of inhibitors targeting mTOR, MEK, PI3K, and PARP, thereby providing diversified combination strategies to overcome therapeutic resistance [77].

Currently, exploration of upstream and downstream nodes as well as bypass signaling pathways within the CDK4/6 axis has provided a foundation for novel drug development. Studies have demonstrated that newly developed CDK2-targeted inhibitors not only effectively induce senescence and apoptosis in ovarian cancer cells with CCNE1 amplification, but also exhibit significant potential in overcoming acquired resistance to CDK4/6 inhibitors [79]. Conversely, miR-506 has been shown to effectively inhibit ovarian cancer cell proliferation and induce senescence by directly targeting CDK4 and CDK6, thereby providing a novel perspective for microRNA-based cell cycle intervention [80].

### 5.4. Other Drugs

PF-562271 effectively inhibits adhesion and migration of ovarian cancer cells by suppressing phosphorylated focal adhesion kinase (p-FAK) expression and disrupting focal adhesion assembly. Additionally, this compound induces G1 phase cell cycle arrest and senescence [81]. Furthermore, various natural products and their derivatives have demonstrated the ability to induce senescence. For instance, the natural compound tanshinone I enhances paclitaxel sensitivity in ovarian cancer cells by promoting DNA damage and inducing cellular senescence [82]. Cardamonin inhibits NF-κB and mTOR pathways to induce G2/M phase arrest and senescence in ovarian cancer cells [83]. Resveratrol derivatives effectively trigger cellular senescence by inducing oxidative stress and DNA damage [84]. Additionally, the traditional Chinese medicine compound Fuling Granules has been demonstrated to mediate cell cycle arrest, apoptosis, and senescence through modulation of the AKT/GSK-3β signaling pathway, thereby exerting anti-ovarian cancer effects [85].

## 6. Metabolic Stress-Induced Ovarian Cancer Cell Senescence

Metabolic stress resulting from rapid tumor cell proliferation represents a critical intrinsic driver of cellular senescence. In ovarian cancer, downregulation of enolase-1 (ENO1) leads to abnormal glucose accumulation, and this metabolic disorder has been demonstrated to correlate directly with senescence levels in cisplatin-resistant cells. Mechanistically, low ENO1 expression promotes cellular senescence and cisplatin resistance by altering p53 and p21 expression patterns [12]. As a classic glucose-regulatory protein, GRP78 has been confirmed to participate in cisplatin-induced ovarian cancer cell senescence through modulation of p53/p21/CDC2 expression and interference with endoplasmic reticulum Ca^2+^ release [6]. Intervention studies have demonstrated that in a murine ovarian cancer model, combined treatment with rosiglitazone and olaparib not only ameliorates the senescent state but also significantly enhances drug sensitivity [16]. Conversely, wild-type IDH1 is highly expressed in high-grade serous carcinomas and is associated with shortened progression-free survival. Pharmacological inhibition or genetic knockdown of IDH1 effectively induces senescence and inhibits proliferation across various HGSC cell lines [16]. Furthermore, targeting another key metabolic enzyme, RRM2, effectively induces senescence in epithelial ovarian cancer cells [86]. In summary, intrinsic metabolic adaptations developed by cancer cells in response to survival pressure constitute a critical driving force for spontaneous senescence.

Stearoyl-CoA desaturase 1 (SCD1) represents a critical hub maintaining lipid metabolism and iron homeostasis. Its inhibition induces endoplasmic reticulum stress, thereby triggering cellular senescence and apoptosis, whereas SCD1 upregulation through lipid desaturation suppresses senescence while concurrently creating conditions for tumor metastasis [13,87]. Iron-sulfur cluster biosynthesis constitutes a crucial step in maintaining cellular homeostasis and preventing senescence. FDX2 deficiency results in iron-sulfur cluster assembly defects, leading to two distinct consequences: dysfunction of iron-sulfur cluster proteins and intracellular iron accumulation. These stressors ultimately converge on p53 pathway activation, thereby inducing cellular senescence [14]. These studies collectively elucidate the central role of lipid metabolism and iron homeostasis in precisely regulating ovarian cancer cell senescence.

Metabolic stress within the tumor microenvironment can also induce ovarian cancer cell senescence through paracrine signaling. Under hypoxic conditions, ovarian cancer tissues can trigger cellular senescence via a MAD2-dependent mechanism [88]. Furthermore, normal cellular components in the tumor microenvironment—such as peritoneal mesothelial cells and fibroblasts—as well as malignant ascites, can induce senescence in primary epithelial ovarian cancer cells through paracrine secretion of factors including GRO-1, HGF, and TGF-β1. This process involves coordinated activation of multiple key signaling pathways, including AKT, AP-1, and ERK1/2, with downstream effects ultimately converging on elevated intracellular reactive oxygen species levels and mitochondrial dysfunction [5]. These external metabolic stresses collectively drive ovarian cancer cells into a senescent state.

## 7. Other Factors Regulating Ovarian Cancer Cell Senescence

Non-classical Wnt signaling can trigger cellular senescence through heterochromatin induction. As a representative ligand of this pathway, Wnt5a activation can initiate the senescence program via two distinct mechanisms: induction of heterochromatin protein 1-γ nuclear aggregation and inhibition of canonical β-catenin signaling. Clinical data further demonstrate that low Wnt5a expression is significantly associated with poor prognosis in ovarian cancer patients [89]. These findings indicate that the Wnt5a-mediated senescence pathway constitutes a natural barrier against tumor progression in vivo.

Tumor-suppressive microRNAs, including miR-519a-5p, miR-512-3p, and miR-143-3p, have been detected in extracellular vesicles (EVs) derived from placental tissue. These microRNAs ultimately induce γ-H2AX accumulation and NF-κB pathway activation by inhibiting expression of their downstream target genes, thereby jointly promoting tumor cell senescence and death [90]. This discovery provides a theoretical foundation and experimental basis for developing natural extracellular vesicles as novel anti-tumor therapeutic vehicles.

## 8. Therapeutic Benefits of Ovarian Cancer Cell Senescence

**Figure 3:**
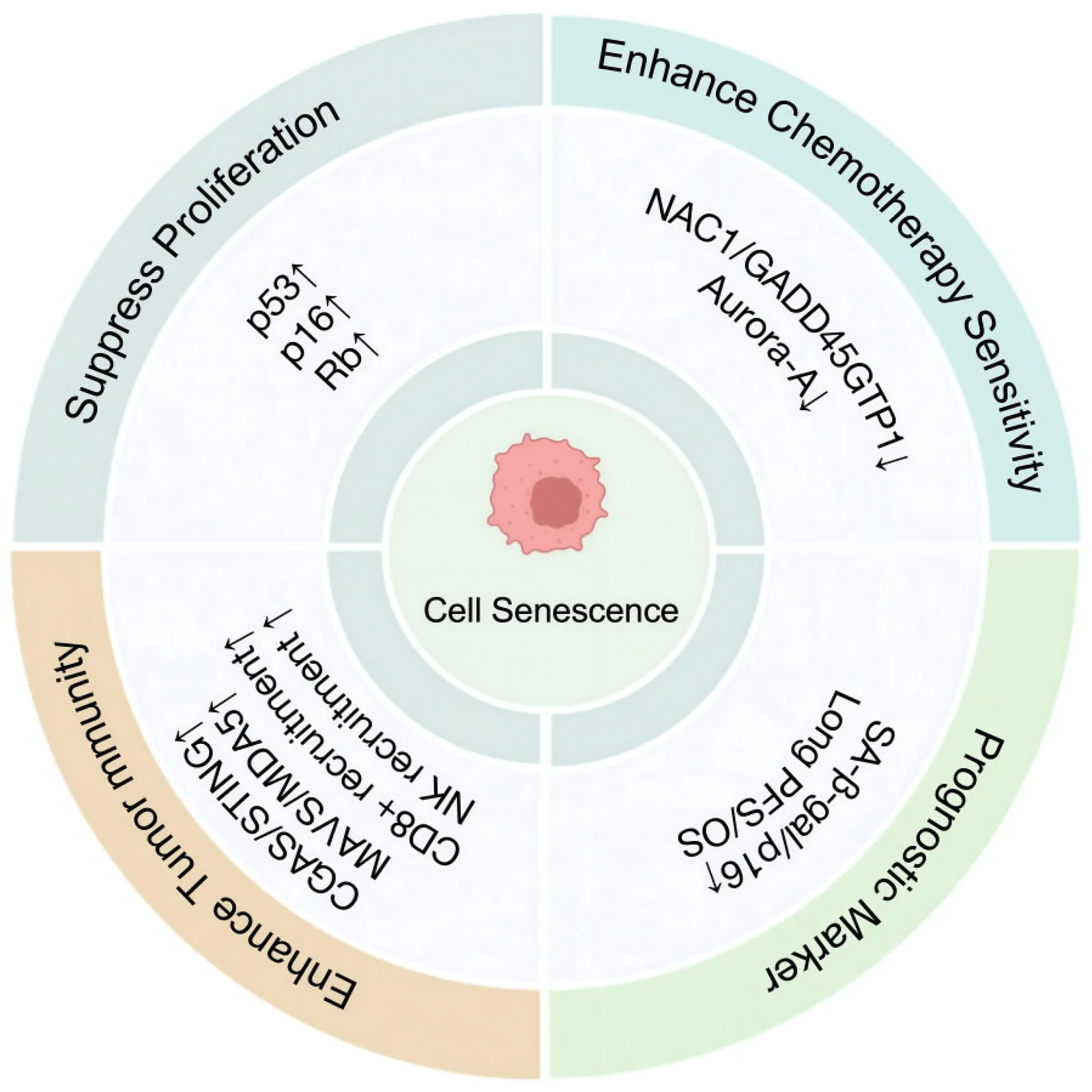
Therapeutic benefits of cell senescence in the treatment of ovarian cancer. Inhibition of proliferation: Upregulation of p53/p16/Rb expression promotes the senescence of ovarian cancer cells and inhibits their proliferation; Enhancing chemosensitivity: Inhibition of the Aurora-A and NAC1/GADD45GIP1 signaling axis can promote the senescence of ovarian cancer cells and enhance chemosensitivity; Activating anti-tumor immunity: Senescent tumor cells secrete SASP, which can promote the infiltration of CD8^+^ T cells and NK cells into tumors by activating the cGAS/STING signaling axis and the MAVS/MDA5 pathway; Prognostic markers: Post-treatment levels of senescence-associated features, including SA-β-gal activity and p16 expression, have been reported to correlate with progression-free survival and overall survival.

Treatment-induced senescence directly inhibits tumor growth by triggering stable proliferative arrest. Studies have demonstrated that various clinically relevant agents—including carboplatin/paclitaxel combination regimens, PARP inhibitors, and CDK4/6 inhibitors—can induce ovarian cancer cell senescence by activating the p16/Rb or p53 pathways, thereby achieving effective anti-proliferative effects [15,72,76] (Figure 3). This senescence-mediated growth arrest provides a crucial therapeutic window for controlling tumor progression. Consequently, inducing cancer cell senescence has emerged as a highly promising anti-cancer strategy distinct from apoptosis, demonstrating significant value in DNA-damaging chemotherapy and targeted therapy.

Treatment-induced senescence enhances chemosensitivity in ovarian cancer. For instance, the natural compound tanshinone I significantly increases paclitaxel sensitivity by promoting DNA damage and senescence [82]. Furthermore, inhibition of the Aurora-A and NAC1/GADD45GIP1 signaling axis relieves senescence suppression, thereby reversing chemoresistance in ovarian cancer organoids and cell lines [17,91].

Specific senescence-associated secretory phenotype components can activate anti-tumor immunity, rendering senescent tumor cells more susceptible to immunotherapy. Studies have demonstrated that PARP inhibitors promote SASP factor release, thereby recruiting immune cells and providing a potential strategy for activating the tumor immune microenvironment [74]. Further mechanistic studies revealed that platinum drugs induce senescent ovarian cancer cells to secrete specific SASP components, which drive type I interferon production through cGAS/STING pathway activation, thereby promoting CD8^+^ T cell and NK cell infiltration into tumors and converting “immunologically cold” tumors to an immunotherapy-sensitive state [92]. Additionally, SMARCA4 inhibition by PROTAC drugs enhances SASP secretion in senescent cells while activating cGAS/STING and MAVS/MDA5 pathways, significantly improving CD8^+^ T cell- and NK cell-mediated immune surveillance [23]. Concurrently, the BET inhibitor i-BET151, during senescence induction, synergistically promotes CD8^+^ T cell infiltration, triggers immunogenic cell death, and inhibits tumor metastasis [93]. These findings collectively demonstrate that drug-induced senescence not only directly inhibits tumor proliferation but also reshapes the tumor immune microenvironment through SASP remodeling, providing novel strategies for combination immunotherapy.

The intrinsic susceptibility of ovarian cancer cells to undergo therapy-induced senescence may represent a potential biomarker for predicting chemotherapy response and long-term patient outcomes. A study utilizing a novel in situ murine model demonstrated that homologous recombination-deficient ovarian cancer exhibited enhanced platinum sensitivity, which was associated with increased induction of senescence-related responses following treatment [92]. In tumor specimens from platinum-treated ovarian cancer patients, chemotherapy was accompanied by increased expression of senescence-associated features, including SA-β-gal activity and p16 positivity. Importantly, higher levels of SA-β-gal-positive cells were associated with improved treatment response, prolonged progression-free survival, and extended overall survival [94,95]. These findings suggest that chemotherapy-induced senescence-associated features may serve as potential predictive and prognostic biomarkers in ovarian cancer. However, because no single marker is sufficient to define cellular senescence, comprehensive evaluation incorporating additional parameters, such as persistent proliferative arrest, DNA damage responses, and SASP profiles, is required for accurate characterization of therapy-induced senescence.

## 9. Detrimental Effects of Ovarian Cancer Cell Senescence in Treatment

**Figure 4:**
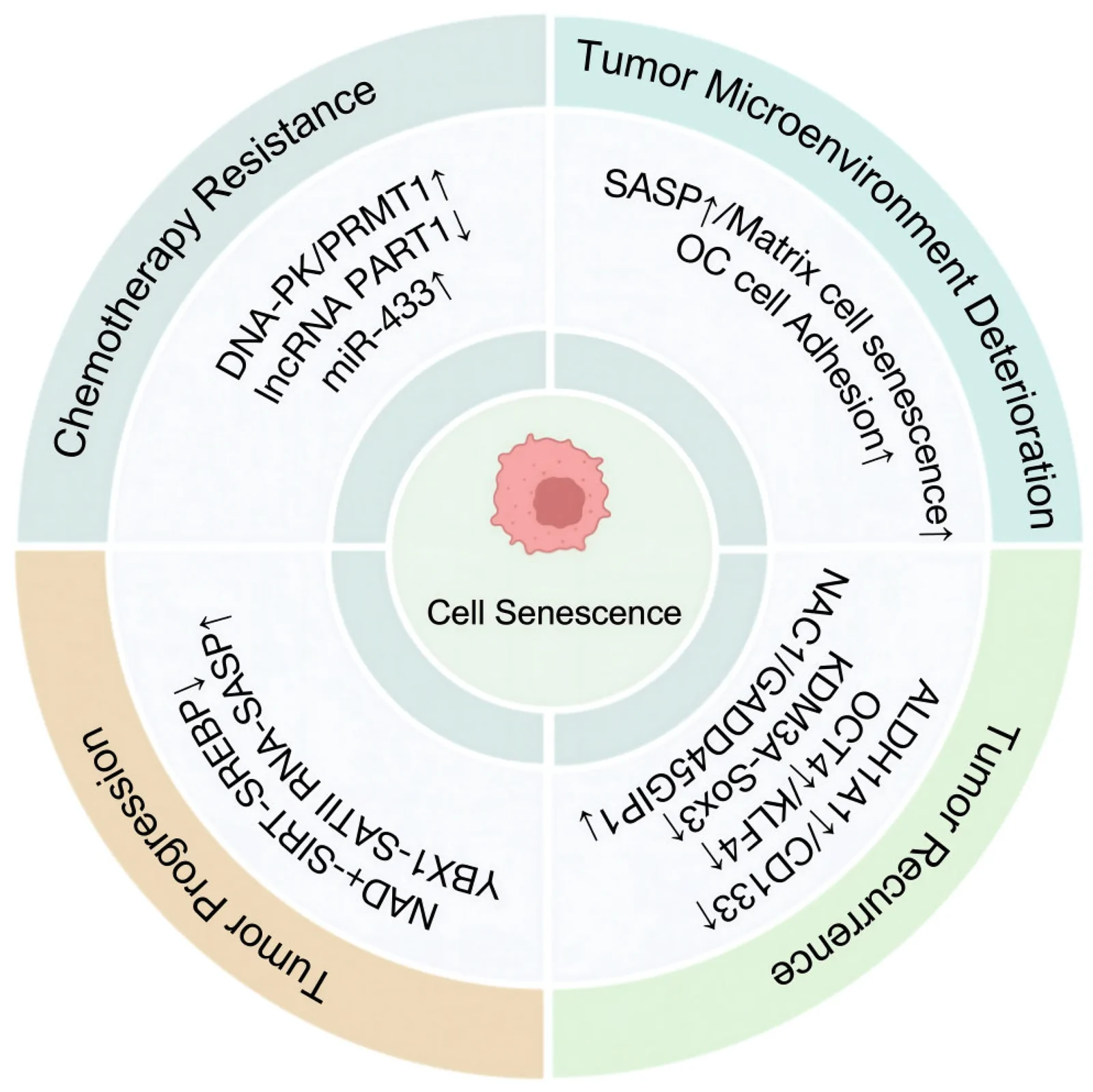
The negative effect of cell senescence in the treatment of ovarian cancer. Chemotherapy resistance: Downregulation of lncRNA PART1, upregulation of miR-433, and DNA-PK/PRMT1 signaling axis activation in senescent ovarian cancer cells mediate therapeutic resistance. Tumor recurrence: High expression of stem cell markers (ALDH1A, CD133, OCT4, KLF4, Sox2) in senescent tumor cells correlates with recurrence; NAC1/GADD45GIP1 axis inhibition suggests senescence reversal. Tumor progression promotion: SASP secreted by ovarian cancer cells modulates the NAD^+^-SIRT1-SREBP axis in adjacent cells, activates the YBX1-SATII RNA axis, and promotes tumor progression and metastasis. Tumor microenvironment deterioration: SASP secreted by senescent ovarian cancer cells induces peritoneal mesothelial cell senescence and promotes tumor adhesion.

Senescent ovarian cancer cells can develop therapeutic resistance through cell cycle arrest. Studies have demonstrated that olaparib-induced senescent cells impair mitochondrial autophagy via downregulation of long non-coding RNA PART1, thereby acquiring PARP inhibitor resistance [75]. Furthermore, miR-433 overexpression directly confers paclitaxel resistance by inducing the senescent phenotype [9]. This senescence-associated cell cycle arrest promotes drug resistance through multiple mechanisms: first, the senescent state itself serves as a cell survival strategy, enabling cancer cells to evade chemotherapeutic killing, representing a key mechanism for long-term resistance [16,96]; second, activation of the DNA-PK/PRMT1 signaling axis maintains senescence-associated secretory phenotype production, creating a microenvironment conducive to tumor cell survival [97] (Figure 4). Intervention studies confirmed that rosiglitazone-mediated amelioration of the senescent state significantly enhances tumor cell chemosensitivity [16].

Therapy-induced senescent ovarian cancer cells can partially acquire stem cell-like characteristics, serving as a critical source for tumor recurrence. Upon maturation of permissive microenvironmental conditions, dormant senescent stem cells can regain proliferative capacity. Studies have demonstrated that chemotherapy-induced senescence constitutes an important driving force for cancer stem-like cell generation. For instance, in cisplatin-treated senescent ovarian cancer cells, NAMPT-mediated metabolic reprogramming promotes manifestation of stem cell characteristics, including high ALDH1A1 expression and CD133 positivity [21]. Similarly, docetaxel-induced multi-drug-resistant polyploid giant cancer cells highly express stem cell markers such as OCT4 and KLF4 [71]. These senescence-associated stem cell populations represent key sources of tumor recurrence and drug resistance. Once the microenvironment becomes permissive, they can resume proliferation through non-classical division mechanisms such as polyploid giant cancer cell budding, ultimately leading to disease recurrence. NAC1 serves as a critical molecule maintaining stem cell characteristics and mediates cisplatin resistance through the NAC1/GADD45GIP1 axis. Notably, NAC1 expression is negatively correlated with ovarian cancer cell senescence, potentially representing a mechanism whereby senescent stem cells reverse senescence and regain proliferative capacity [17]. Furthermore, KDM3A is abundantly expressed in human ovarian cancer tissues and positively correlated with stem cell marker Sox2 expression and chemoresistance [38].

SASP secreted by senescent cancer cells can stimulate proliferation, invasion, and epithelial mesenchymal transition of adjacent cancer cells through paracrine signaling [18]. Clinical dataset analysis reveals elevated YBX1 expression in cancer-associated stroma of breast and ovarian cancers compared with normal stroma, correlating with poor prognosis. YBX1 promotes SATII RNA packaging into small extracellular vesicles, which can induce cellular senescence and upregulate inflammatory SASP genes in recipient cells [98]. Recent studies demonstrate that SASP inhibits plasma membrane cholesterol reduction through NAD^+^-SIRT1-SREBP axis modulation in adjacent cancer cells, thereby promoting tumor cell detachment and metastasis independent of proliferation [99].

SASP can induce stromal cell senescence through tumor microenvironment remodeling, thereby creating a pro-metastatic niche conducive to tumor progression. Studies have shown that ovarian cancer cell-conditioned medium inhibits human peritoneal mesothelial cell proliferation and induces senescence, thereby enhancing tumor cell adhesion. Clinical sample analysis revealed abundant senescent mesothelial cells in peritumoral regions, confirming this process at the pathological level [100]. Compared with drug-induced senescence, spontaneous senescence of ovarian cancer cells exhibits stronger tumorigenic potential, more effectively promoting angiogenesis, stromal remodeling, and tumor growth in vivo [95].

## 10. The Application of Targeting Ovarian Cancer Cell Senescence in Treatment

To address clinical challenges of chemoresistance and high recurrence rates in ovarian cancer, the scientific community is actively exploring novel therapeutic paradigms targeting tumor cell senescence. Developing multi-dimensional treatment strategies capable of precisely inducing senescence, selectively eliminating senescent cells, and effectively neutralizing deleterious SASP constituents represents the key to overcoming current therapeutic bottlenecks. To facilitate comparison among different senescence-oriented therapeutic strategies, we summarize representative approaches, their molecular mechanisms, experimental evidence, and current translational status in Table 1.

### 10.1. Targeted Epigenetic Regulatory Factors

DNA methyltransferase inhibitors, including 5-aza-2′-deoxycytidine (5-AZA-dC), can alleviate epigenetic silencing of cell cycle inhibitory genes such as p21 and effectively induce ovarian cancer cell senescence [37]. Multiple studies have confirmed that 5-AZA-dC regulates expression of key genes through DNA demethylation, including downregulation of TGFBI [101] and CCDC69 [102], thereby enhancing platinum sensitivity in ovarian cancer cells. The demethylating agent zebularine reduces the cisplatin IC_50_ in resistant cell line A2780/CP by 16-fold, achieving resensitization of drug-resistant cells [103]. Furthermore, combination of 5-AZA-dC with everolimus (RAD001) more significantly inhibits formation of ovarian clear cell carcinoma stem/progenitor cells and spheroids compared with monotherapy. This synergistic effect involves inhibition of the COL6A3-AKT-mTOR signaling pathway [104]. Notably, although 5-AZA-dC primarily induces senescence and apoptosis in ovarian cancer cells, it may also enhance migratory capacity to some extent through upregulation of glycosyltransferases including MGAT5 and ST3GAL4 [37].

Histone deacetylase inhibitors, including trichostatin A and sodium butyrate, can induce senescence-like phenotypes in G0/G1 phase through p21-mediated pRb dephosphorylation, and this effect remains effective even in cancer cells with p53 or Rb dysfunction [41,105]. Conversely, histone methyltransferase EZH2 has been reported to suppress p53-mediated senescence pathways through H3K27me3-dependent chromatin regulation in several cancer models. These findings suggest that EZH2-mediated epigenetic regulation may contribute to senescence resistance and provide a mechanistic rationale for exploring EZH2-targeting approaches as potential combination strategies in ovarian cancer, although direct validation in ovarian cancer models remains limited [8]. The EZH2 inhibitor 3-deazaneplanocin A (DZNep) has been demonstrated in colon cancer models to inhibit HCT116 cell proliferation through induction of senescence and apoptosis [106]. Similarly, the specific EZH2 inhibitor GSK126 has been reported to induce senescence and exert anti-tumor effects in multiple myeloma cells both in vitro and in vivo [107]. These findings from other malignancies suggest that EZH2-mediated epigenetic regulation may represent a conserved mechanism involved in senescence control. However, whether EZH2 inhibition directly induces senescence in ovarian cancer remains insufficiently validated and requires further investigation. Additionally, protein arginine methyltransferase 1 (PRMT1) is phosphorylated by DNA-PK and recruited to chromatin during cisplatin-induced DNA damage response, promoting SASP production through histone H4R3 methylation; in vitro studies have shown that PRMT1 inhibition enhances cancer cell chemosensitivity [97]. Recent studies have also identified lysine demethylase KDM3A as a regulator of multiple targets including Sox2, Nanog, Bcl-2, and p21, participating in maintenance of tumor stemness, proliferation regulation, and chemoresistance; its inhibition significantly controls tumor growth and reduces recurrence risk [38].

Non-coding RNAs participate in senescence regulation through complex networks. Studies have identified certain senescence-associated long non-coding RNAs that regulate mitochondrial metabolism through DLST protein binding, and their knockdown restores cisplatin chemosensitivity in ovarian cancer cells [45]. Recent studies demonstrate that blockade of the lncRNA-PART1-PHB2 axis promotes ovarian cancer cell senescence and enhances PARP inhibitor sensitivity [75]. At the microRNA level, miR-493-3p overexpression induces mitotic abnormalities and promotes cellular senescence [47], whereas miR-34 family inactivation in ovarian cancer synergizes with p53 mutations to promote tumorigenesis [7]. Furthermore, restoration of miR-34 expression in leukemia stem cells has been shown to activate senescence-associated programs and exert anti-tumor effects [108]. Although miR-34 dysregulation has also been reported in ovarian cancer, its role in regulating senescence in this context remains unclear.

### 10.2. Targeted Metabolic Regulatory Factors

Metabolic reprogramming constitutes a key mechanism whereby ovarian cancer cells respond to therapeutic stress and develop drug resistance. Targeted modulation of senescence-associated metabolic pathways represents a promising research direction for overcoming chemoresistance in ovarian cancer.

Targeting NAD^+^ metabolism and the ALDH pathway enables selective elimination of senescence-associated tumor stem cell subpopulations. NAMPT, the key rate-limiting enzyme in NAD^+^ biosynthesis, can be inhibited by FK866, which, when combined with platinum-based chemotherapy, effectively eliminates cisplatin-induced senescence-associated cancer stem cells, demonstrating synergistic anti-tumor effects both in vitro and in vivo [21]. Similarly, the ALDH1A1 inhibitor compound 974 significantly blocks platinum-induced senescence and stemness manifestations [22]. Furthermore, wild-type IDH1, a critical molecule in energy metabolism, promotes tumorigenesis through epigenetic mechanisms; its inhibition induces cellular senescence by increasing the repressive histone mark H3K9me2 and downregulating E2F target genes [42]. Collectively, targeting key energy metabolic vulnerabilities represents a potential approach for developing combination strategies. However, current evidence remains largely preclinical, and further validation is required to determine whether metabolic interventions can effectively improve therapeutic outcomes in ovarian cancer.

Ferroptosis induction has emerged as a potential strategy for targeting senescence-associated metabolic vulnerabilities. However, current evidence is mainly derived from experimental models. The resveratrol derivative 3,3′,4,4′-tetrahydroxystilbene (THS) induces cellular senescence and apoptosis by disrupting redox homeostasis and activating the p38 MAPK pathway [84]. Recent studies demonstrate that spinach polysaccharide induces ACSL4-dependent ferroptosis in ovarian cancer cells through accumulation of lipid reactive oxygen species, Fe^2+^, and malondialdehyde, concomitant with reduced glutathione production [109]. Concurrently, novel multi-target heterocyclic ferroquinone compounds exhibit potential for overcoming cisplatin resistance through induction of oxidative stress and senescence pathways [110]. Notably, studies have revealed that therapy-induced senescent ovarian cancer cells undergo unique iron metabolism reprogramming, resulting in intracellular iron overload and consequent specific sensitivity to ferroptosis. This discovery provides a rationale for utilizing ferroptosis inducers to selectively eliminate senescent cells refractory to conventional senolytic agents [24].

### 10.3. SASP Modulation

Given the dual role of SASP in ovarian cancer therapy—enhancing anti-tumor immune surveillance while also promoting tumor progression and therapeutic resistance—SASP-targeted interventions represent a promising but complex strategy. Importantly, SASP is a highly dynamic secretory program rather than a uniform phenotype, with its composition and function shaped by the tumor microenvironment and intrinsic cellular state [3,111].

The magnitude and quality of SASP are influenced by multiple factors, including the type of senescence-inducing stimulus (e.g., chemotherapy, oncogene activation, or DNA damage), tumor genetic background (such as p53 and NF-κB status), the cellular origin of senescent cells, and whether senescence is transient or persistent. Additional modulators, such as treatment duration, metabolic reprogramming, hypoxia, and immune cell infiltration, further determine whether SASP supports immune-mediated tumor clearance or drives a pro-tumor inflammatory milieu [112].

Mechanistically, SASP can be divided into two functional states. An acute, immune-stimulatory SASP is associated with early or transient senescence and promotes immune cell recruitment and activation, facilitating senescent cell clearance and tumor [2]. In contrast, a chronic SASP arises in persistent senescent cells and is enriched in pro-inflammatory and pro-tumorigenic factors that promote angiogenesis, matrix remodeling, immune evasion, and therapeutic resistance [113]. This dichotomy explains why global SASP inhibition may be detrimental, as it could also impair beneficial immune surveillance functions [2].

In ovarian cancer, emerging evidence suggests that inflammatory signaling pathways associated with SASP components may contribute to chemotherapy resistance and represent potential targets for therapeutic intervention. Tumor microenvironment-derived soluble factors, particularly interleukin-6 (IL-6), have been implicated in promoting platinum resistance through activation of the JAK/STAT signaling pathway. Previous studies demonstrated that conditioned medium derived from tumor-activated stromal cells enhanced platinum resistance in ovarian cancer cells, partly through soluble inflammatory mediators including IL-6, and pharmacological inhibition of JAK signaling partially restored platinum sensitivity [114]. Given that IL-6 is a major component of the SASP, these findings provide a mechanistic rationale suggesting that targeting JAK-mediated inflammatory signaling may attenuate SASP-associated tumor-promoting effects, although direct evidence demonstrating SASP modulation in senescent ovarian cancer cells remains limited.

Supporting its translational relevance, a randomized phase I/II clinical study evaluated the JAK1/2 inhibitor ruxolitinib in combination with standard paclitaxel/carboplatin chemotherapy in patients with newly diagnosed advanced ovarian, fallopian tube, or primary peritoneal carcinoma receiving neoadjuvant treatment [115]. The combination regimen was tolerable and showed a trend toward improved progression-free survival compared with chemotherapy alone (median PFS: 14.6 versus 11.6 months; HR = 0.702, *p* = 0.059). However, this clinical study did not include pharmacodynamic analyses of SASP components or senescence-associated biomarkers. Future studies integrating SASP profiling and senescence-related biomarkers are required to determine whether JAK inhibition can selectively suppress detrimental inflammatory SASP programs while preserving beneficial immune surveillance functions.

### 10.4. Senolytics

The anti-aging protein Klotho effectively inhibits tumor progression by interfering with multiple oncogenic signaling pathways. In ovarian cancer, Klotho expression is significantly downregulated at both tissue and cell line levels, and this downregulation is associated with wild-type BRCA status [116]. Klotho overexpression not only directly inhibits cancer cell proliferation and migration but also exerts broad anti-tumor effects through simultaneous blockade of IGF-1 and estrogen receptor signaling pathways [116,117]. Restoration of Klotho expression in preclinical models significantly enhances cisplatin chemosensitivity in ovarian cancer cells [118].

Senolytic agents, which directly eliminate senescent cells, demonstrate significantly enhanced anti-tumor efficacy and delayed drug resistance development when combined with conventional therapies. Studies have demonstrated that PARP inhibitor-induced polyploid giant cancer cells can generate drug-resistant progeny through non-classical division mechanisms. Mifepristone effectively blocks this process. Combination of mifepristone with olaparib significantly inhibits tumor growth, even in resistant models, whereas mifepristone monotherapy effectively reduces tumor burden [119]. Furthermore, the classic senolytic combination of dasatinib and quercetin, when combined with carboplatin or olaparib, effectively inhibits ovarian cancer metastasis to peritoneal and adipose tissues and significantly improves survival outcomes in tumor-bearing mice [20]. These studies suggest that senolytic-based combination approaches represent a potential strategy for selectively eliminating therapy-induced senescent cells and enhancing the efficacy of anticancer treatments. However, current evidence is primarily derived from preclinical models, and further studies are required to determine their therapeutic applicability in ovarian cancer.

### 10.5. Other

Various natural compounds effectively induce ovarian cancer cell senescence through modulation of distinct core signaling pathways. Phellinus linteus extract regulates the AKT/GSK-3β pathway to induce cell cycle arrest, apoptosis, and senescence while inhibiting epithelial mesenchymal transition, demonstrating broad-spectrum anti-tumor activity in both in vitro and in vivo models [85]. Mumefural exhibits cell-specific effects, inducing G1 and G2/M phase arrest and senescence phenotypes in ovarian cancer cells with specific genetic backgrounds through RSK phosphorylation inhibition; its efficacy correlates closely with p16 and p53 status [120]. Punicic acid effectively induces cellular senescence through activation of p53-mediated DNA damage response and generates synergistic anti-tumor effects with carboplatin in preclinical studies, exhibiting promising translational potential [121].

## 11. Issues and Prospects

This review systematically synthesizes key advancements in ovarian cancer cellular senescence research, highlighting its dual, complex role in therapy as both tumor-suppressive and tumor-promoting. Research indicates that telomere dysfunction, epigenetic reprogramming, dysregulated hormonal signaling, metabolic stress, and the tumor microenvironment collectively constitute a finely regulated network that coordinately induces and maintains senescence in ovarian cancer cells. Therapeutically, senescence exerts anti-tumor effects through mechanisms including irreversible cell cycle arrest, serving as a predictor of chemosensitivity, and activating anti-tumor immunity via SASP. However, the senescent state represents a double-edged sword: certain senescent cells can mediate therapeutic resistance, tumor recurrence, and disease progression through entry into dormancy, acquisition of stem-like properties, or secretion of pro-tumorigenic SASP components. The ultimate biological outcome depends critically on the context of senescence induction, the tumor mutational landscape, and dynamic evolution of the tumor microenvironment.

Despite significant progress, several critical challenges remain before senescence-oriented therapies can be translated into clinical practice. First, reliable approaches are needed to identify and distinguish functionally distinct senescent populations, including therapy-induced senescent tumor cells, senescent stromal cells, and other stress-associated cellular states. Because no single biomarker can comprehensively define senescence, integrated assessment incorporating multiple senescence-associated features, SASP profiles, and microenvironmental characteristics will be essential for accurate patient stratification. Second, the heterogeneity of SASP represents a major barrier to therapeutic application. Future strategies should aim to selectively suppress tumor-promoting inflammatory SASP programs while preserving immune-stimulatory signals that facilitate immune-mediated clearance of senescent cells.

Moreover, the optimal therapeutic sequence between senescence induction and senescent cell elimination requires further investigation. Rather than simply increasing or eliminating senescent cells, future interventions should consider the timing, duration, and cellular context of senescence to maximize anti-tumor efficacy while minimizing adverse effects. Biomarker-guided treatment selection will likely be required to identify patients who are most likely to benefit from senescence-modulating approaches. Importantly, the potential impact of these therapies on normal senescent cells involved in tissue repair, wound healing, and physiological homeostasis must also be carefully evaluated.

Finally, successful clinical translation will require rigorous validation in clinically relevant models, including patient-derived organoids, immune-competent models, and humanized systems that better recapitulate tumor heterogeneity and immune interactions. Future research should therefore shift from broad induction or elimination of senescence toward precision senescence modulation, integrating senescence biomarkers, SASP profiling, immune contexture, and rational combination strategies with chemotherapy, targeted therapy, or immunotherapy. Such a framework may provide new opportunities to overcome chemoresistance and prevent recurrence in ovarian cancer.

## Figures and Tables

**Table 1:** Therapeutic strategies targeting cellular senescence in ovarian cancer: mechanisms, evidence, and translational status.

Time	Representative Agents	Molecular mechanism Related to Senescence	Evidence	Translational Status
Chemotherapy-induced senescence	Carboplatin, cisplatin	DNA damage → DDR activation → p53/p21, p16/Rb activation	Ovarian cancer models and patient samples	Established clinical treatment; senescence consequence under investigation
PARP inhibition	Olaparib, niraparib	Replication stress, DNA damage accumulation, senescence induction	BRCA-mutated ovarian cancer models	Clinically approved targeted therapy
CDK4/6 inhibition	Palbociclib, ribociclib, abemaciclib	RB pathway activation → G1 arrest and senescence-like phenotype	Preclinical ovarian cancer studies	Clinical trials in selected settings
EZH2 inhibition	DZNep, GSK126	Epigenetic reprogramming, activation of senescence pathways	Mainly other cancer models; potential relevance to ovarian cancer	Preclinical
SASP modulation	JAK inhibitors (ruxolitinib)	Suppression of IL-6/JAK/STAT-mediated SASP signaling	Ovarian cancer models and early clinical trials	Early clinical investigation
Senolytic therapy	BCL-2 inhibitors, FOXO4-DRI, etc.	Selective elimination of senescent cells	Mainly preclinical cancer models	Preclinical
Metabolic targeting	NAMPT inhibitors, metabolic modulators	Disrupt survival pathways of senescent cells	Preclinical studies	Preclinical
Ferroptosis induction	GPX4 inhibitors, etc.	Metabolic vulnerability of senescent cancer cells	Emerging evidence	Preclinical

## Data Availability

No new data were created or analyzed in this study. Data sharing is not applicable to this article.
